# A high-density linkage map and fine QTL mapping of architecture, phenology, and yield-related traits in faba bean (*Vicia faba* L.)

**DOI:** 10.3389/fpls.2025.1457812

**Published:** 2025-04-07

**Authors:** David Aguilar-Benitez, Natalia Gutierrez, Inés Casimiro-Soriguer, Ana M. Torres

**Affiliations:** Área de Mejora Vegetal y Biotecnología, Instituto de Investigación y Formación Agraria, Pesquera, Alimentaria y de la Producción Ecológica (IFAPA) Centro “Alameda del Obispo”, Córdoba, Spain

**Keywords:** faba bean, density map, QTLs, autofertility, flowering time, plant architecture, yield, dehiscence

## Abstract

Faba bean is a key protein feed and food worldwide that still requires accurate genomic tools to facilitate molecular marker-assisted breeding. Efficient quantitative trait locus (QTL) mapping in faba bean is restricted by the low or medium density of most of the available genetic maps. In this study, a recombinant inbred line faba bean population including 124 lines from the cross Vf6 x Vf27, highly segregating for autofertility, flowering time, plant architecture, dehiscence, and yield-related traits, was genotyped using the ‘Vfaba_v2’ SNP array. Genotypic data were used to generate a high-density genetic map that, after quality control and filtering, included 2,296 SNP markers. The final map consisted of 1,674 bin markers distributed across the six faba bean chromosomes, covering 2,963.87 cM with an average marker distance of 1.77 cM. A comparison of the physical and genetic maps revealed a good correspondence between chromosomes and linkage groups. QTL analysis of 66 segregating traits, previously phenotyped in different environments and years, identified 99 significant QTLs corresponding to 35 of the traits. Most QTLs were stable over the years and QTLs for highly correlated traits were mapped to the same or adjacent genomic regions. Colocalization of QTLs occurred in 13 major regions, joining three or more overlapping QTLs. Some of the pleiotropic QTL regions, especially in chromosome VI, shared the same significant marker for different traits related to pollen quantity and size, number of ovules per ovary, seeds per pod, and pod set. Finally, several putative candidate genes for yield-related traits, recently identified using a genome-wide association study, fall inside the colocalizing groups described in this study, indicating that, apart from refining the position of the QTLs and the detection of candidates, the dense new map provides a valuable tool for validation of causative loci derived from association studies and will help advance breeding programs in this crop.

## Introduction

Faba bean (*Vicia faba* L.), one of the oldest and most globally grown cool-season grain legumes, is a partially allogamous insect-pollinated species with a mixed mating system. This legume ranks sixth in terms of the world average production of pulse crops (FAOSTAT, 2022) with a value of 6.1 Mt. China is the largest producer, followed by Ethiopia, the United Kingdom, Australia, and France. With one of the highest protein contents, faba bean is a major food and feed crop used worldwide as a dry grain (pulse), green grain/pod, and green manure. Apart from its nutritional value, faba bean cultivation contributes to more sustainable production in the entire cropping system by providing several agronomic, environmental, and ecological services. By fixing atmospheric nitrogen in symbiosis with rhizobia, faba bean improves soil fertility and contributes to a reduction in the use of synthetic fertilizers, thus promoting circularity and mitigating the environmental impact associated with N_2_O emissions. Moreover, faba bean crop rotations promote diversification, improves the yield of the subsequent crop, and breaks the cycles of pests and diseases, enhancing soil health and structure.

Despite the global significance of the crop and its long history of use, from the 60s to the present, faba bean worldwide cultivation has decreased from 5.4 million hectares to approximately 2.7 million hectares (FAOSTAT, 2022). Lower faba bean profitability compared to other cash crops (such as cereals) and the instability of yields are among the main causes of this decline (Zander et al., 2016). Faba bean production is often threatened by environmental conditions, especially extreme temperatures, drought, and acidity, that, together with diseases (e.g. chocolate spot or ascochyta blight), parasitic weeds of the *Orobanche* genus, and pests (e.g. leaf or seed weevils and aphids), reduce crop yield and affect the commercialization of the grains. Therefore, resistance/tolerance to these biotic and abiotic stresses, together with yield and quality trait improvements, are major priorities in current breeding programs.

Valuable progress has been made through conventional breeding in the last century but faba bean genomic studies have lagged far behind other grain legume crops. One limitation for such development in faba bean has been its large and complex genome (13 Gbp), the largest among the cool season legumes, with a high presence of repeat DNA (Macas et al., 2015), which has hampered the development of saturated genetic linkage maps and the development of diagnostic markers within the quantitative trait locus (QTL) intervals. In the last two decades, however, significant efforts have been made to enrich genetic and genomic resources in this crop, and faba bean has also slowly benefited from modern breeding methods based on molecular genetic tools (Adhikari et al., 2021; Khazaei et al., 2021). As a result, a large number of markers and maps have now been developed that can be used to understand faba bean genetics and in comparative genomic studies.

The first genetic maps were based on morphological and isoenzyme markers, anonymous random amplified polymorphic DNA (RAPD) markers, and simple sequence repeats (SSR) (Torres et al., 1993; Vaz Patto et al., 1999; Román et al., 2002; Avila et al., 2004; Román et al., 2004; Avila et al., 2005; Ellwood et al., 2008; Zeid et al., 2009; Ma et al., 2013). RAPDs were further used to develop trait-linked sequence characterized amplified regions (SCARs) for simple seed quality parameters such as tannins and vicine-convicine (Gutierrez et al., 2006, 2007, 2008) or for the selective selection of the determinate growth habit (Avila et al., 2006, 2007). Subsequently, comparative genomic approaches with the model *Medicago truncatula* and the development of high-throughput sequencing technologies allowed the development of expressed sequence tags (ESTs) and the identification of single nucleotide polymorphisms (SNPs) that reinforced faba bean genetic studies and breeding approaches (Kaur et al., 2014; Ocaña et al., 2015; Ray et al., 2015; Webb et al., 2016; Carrillo-Perdomo et al., 2020).

Biparental mapping populations have been used in faba bean to identify QTLs associated with disease resistances, such as ascochyta and broomrape resistance (Román et al., 2002, 2003; Avila et al., 2004, 2005; Díaz-Ruiz et al., 2009, 2010; Gutierrez et al., 2013; Kaur et al., 2014; Atienza et al., 2016; Ocaña-Moral et al., 2017; Sudheesh et al., 2019; Gutierrez and Torres, 2021); abiotic stresses (Arbaoui et al., 2008); and architectural, flowering and yield-related traits in different genetic backgrounds and populations (Cruz-Izquierdo et al., 2012; Avila et al., 2017; Catt et al., 2017). Furthermore, consensus maps have integrated the information of multiple populations from diverse genetic backgrounds. The first consensus map in faba bean was reported by Satovic et al. (2013) with 729 markers, half of them being RAPDs. Subsequently, Webb et al. (2016) developed the first consensus map based exclusively on SNPs derived from *M. truncatula*, which contained 687 markers using six mapping populations. Moreover, Carrillo-Perdomo et al. (2020) reported a high saturated faba bean consensus map with 1,728 SNP markers, using three mapping populations.

The development of dense and robust genetic maps based on gene-based markers is a prerequisite for gene mapping, gene cloning, and marker-assisted selection (MAS). The availability of large-scale SNP markers, together with the reduction in sequencing and genotyping cost, has enabled the development of cost-effective low- to high-density genotyping platforms for applied genomic research outside of model species (Zhao et al., 2023). One of the first faba bean high density genotyping platforms combined information from different faba bean transcriptome datasets (Ocaña et al., 2015; Webb et al., 2016; Khan et al., 2019; Lyu et al., 2021) to develop the Vfaba_v2 Axiom SNP array with 60K SNPs (O’Sullivan et al., 2019; Khazaei et al., 2021), which is available from the University of Reading, UK. Recently, Zhao et al. (2023), using the Faba_bean_130 K SNP TNGS genotyping platform, developed the most ultra-dense faba bean map published thus far, consisting of 12,023 SNP markers. These new genomic tools have been utilized to identify alleles associated with target traits using different methods, such as QTL mapping or genome-wide association studies (GWASs) (Sallam et al., 2022). Some of the GWASs reported thus far in this crop aimed to identify candidate genes associated with frost, freezing, heat, and drought tolerance (Ali et al., 2016; Sallam et al., 2016; Maalouf et al., 2022; Sallam et al., 2022; Gutierrez et al., 2023), resistance to *Ascochyta fabae* (Faridi et al., 2021); tolerance to herbicides (Abou-Khater et al., 2022); phenological and morphological traits (Skovbjerg et al., 2023; Jayakodi et al., 2023); flowering and seed quality traits (Ohm et al., 2024); and yield-related traits (Skovbjerg et al., 2023; Gutierrez et al., 2024) such as pod and seeds per plant, hundred seed weight, seed size, and plot yield. Furthermore, the recent development of a faba bean reference genome (Jayakodi et al., 2023) provides a foundational resource for molecular breeding and increases the opportunities to identify the specific genes and alleles that underpin valuable traits. This new genomic tool represents a step-change in the process, allowing the comparison of significant markers for major genes or QTLs detected from both interval maps and GWASs and thus providing more strength of evidence for further validation experiments.

Despite the significant advances described above, the efficiency and precision of QTL mapping in faba bean is still restricted by the low or medium density of most of the available faba bean maps. For this reason, our objective in this study was to re-analyse the phenotypic data accumulated in a recombinant inbred line (RIL) population in which genotyping has been updated using the Vfaba_v2 SNP array to generate a high-density genetic map. Subsequently, QTL mapping for autofertility, flowering time, plant architecture, dehiscence, and yield-related traits, crucial factors for faba bean performance and final production, were conducted. The high-density genetic map and the QTLs detected will be the basis for fine mapping and the identification of candidate genes for future marker-assisted breeding in this crop.

## Materials and methods

### Mapping population

In this study, we used the RIL population Vf6 x Vf27 consisting of 124 F_8:9_ individuals developed from single seed descendants in insect proof cages. The female genotype Vf6 is an asynaptic equina line and the male parent Vf27 is a paucijuga type with small seed size considered to be close to the unknown faba bean wild progenitor. Both lines belong to the Instituto de Investigación y Formación Agraria, Pesquera, Alimentaria y de la Producción Ecológica (IFAPA) germplasm collection. The population segregates for architecture, flowering time, autofertility, dehiscence, and yield-related traits. Phenotypic evaluations for autofertility (33 traits) (Aguilar-Benitez et al., 2022), flowering time (4 traits) (Cruz-Izquierdo et al., 2012; Aguilar-Benitez et al., 2021), plant architecture (15 traits) (Avila et al., 2017), yield (9 traits) (Cruz-Izquierdo et al., 2012; Avila et al., 2017), and dehiscence (5 traits) (Aguilar-Benitez et al., 2020a) were used for QTL mapping and the high density map was built upon the most recent map reported (Aguilar-Benitez et al., 2022). Descriptions of the different traits and the acronyms used are given in **Supplementary Table S1**.

### Genetic map

#### Genotyping and quality control

For DNA extraction, young leaves were collected from each individual plant, frozen, and stored at -80°C until use. Genomic DNA was isolated using a DNeasy Plant Kit (QIAGEN ltd, UK). DNA quality was checked using agarose gel electrophoresis while the concentration was estimated using the QubitTM dsDNA Broad Range Assay Kit (Invitrogen by ThermoFisher Scientific, UK), following the manufacturer’s instructions.

For genotyping, we used the ‘Vfaba_v2’ 60k SNP array (O’Sullivan et al., 2019; Khazaei et al., 2021). Polymorphic SNP markers were incorporated into the previous marker dataset (Aguilar-Benitez et al., 2022) to develop a high-density genetic map. To determine the SNP chromosomal position and their corresponding contig, flanking sequences were aligned against the *Vicia faba* reference genome (Jayakodi et al., 2023) using the “map to reference” option in Geneious v.7.1.9 (Gutierrez et al., 2023). After quality control, only markers with a call rate above 97%, a minor allele frequency (MAF) above 30%, and less than 10% heterozygotes were used for further analysis. Furthermore, individuals showing poor DNA quality were removed. Missing alleles were imputed with the LD-kNNi method (Money et al., 2015) in Tassel v5.2.88 (Bradbury et al., 2007).

#### Genetic map construction

The BIN tool algorithm in QTL IciMapping 4.2 (Meng et al., 2015) was used to bin the markers with identical segregation patterns. Segregated data were analyzed for goodness of fit to the expected 1:1 ratio using the chi-square test, and linkage groups were determined based on the LOD threshold value ranging from 6.5 to 10.5. The ordering of markers distributed over each linkage group was performed using the two-opt heuristic algorithm in the QTL IciMapping software. The visualization of the high density SNP marker map was conducted in R 3.6.1 (R Development Core Team, 2022) using the *LinkageMapView* package (Ouellette et al., 2018). A comparative assembly of the alignment between the genetic and physical maps was obtained using the software implemented in the Pretzel platform (Keeble-Gagnère et al., 2019; http://pulses.plantinformatics.io/mapview).

### QTL analysis

The high-density genetic map was used in the QTL analysis based on prior phenotypic evaluations. The QTLs were named using the trait’s abbreviated name followed by a number when the trait was evaluated in different years. A list of traits used in this study with detailed descriptions is shown in **Supplementary Table S1**. QTL detection was conducted using the maximum likelihood (ML) algorithm in the R package *R/qtl* (Broman et al., 2003), performing a single-QTL genome scan with a normal model. The threshold for the detection of a QTL was determined using 1,000 permutations and a significance (*p*-value) level of 0.05. The most likely position of a QTL was estimated by the point where the maximum LOD score was found. The confidence interval of each QTL position was estimated with the 1-LOD and 2-LOD support intervals (Conneally et al., 1985; van Ooijen, 1992). The percentage of phenotypic variance explained (R^2^) by each QTL was obtained using the method proposed by Stansell et al. (2019). To visualize the position of each QTL, the information of the relative position of the peak of LOD was added to the map.

### Colocalizing groups

Overlapping QTLs within a given genetic region were designed as colocalizing groups. The criteria to determine the colocalizing groups were: (1) a group has to contain at least three QTLs, (2) QTLs with confidence intervals connecting different QTL groups were discarded and, (3) the LOD peak of each QTL within a group has to fall in or very close to the confidence interval of the other colocalized QTLs. To understand the relationship among the traits, we performed a Pearson correlation analysis for each colocalizing group using the *PerformanceAnalytics* R package (Peterson and Carl, 2020).

The physical map information was used to determine the number of genes falling within each QTL or colocalizing group interval and the QTL distance in megabase pairs (Mbp).

### Candidate gene prediction

The sequences flanking each significant SNP marker detected in the QTL analysis or the markers belonging to the same bin were blasted (BLASTx) against the NCBI (https://blast.ncbi.nlm.nih.gov/Blast.cgi) *Medicago truncatula* and *Arabidopsis thaliana* reference genomes to annotate the potential candidate genes responsible of each trait.

## Results

### Genetic map

A population of 124 RILs and their parental lines were genotyped using the ‘Vfaba_v2’ 60k Axiom array, obtaining 34,320 SNP markers with a call rate above 97%. After quality control, seven individual samples were removed from the genotyping matrix due to poor DNA quality. The 34,320 SNP markers and the previous dataset (450 markers) were filtered for MAF > 30% and heterozygotes < 10%, obtaining a final matrix of 2,296 SNP markers.

Co-segregated or tightly linked SNP markers, continuous at non-recombining intervals, were defined as bin markers (Guo et al., 2023). We obtained 1,676 bin markers that were finally used to construct the genetic map. Two of the bin markers were unlinked, and thus, the final map contained 1,674 bin markers well-distributed across the six faba bean chromosomes in eight linkage groups (chromosomes II and V were separated into two linkage groups each). Chromosome assignment of the SNP markers was performed by mapping the sequences against the recent faba bean genomic sequence (Jayakodi et al., 2023). Thus, 1,942 of them could be ascribed to a specific chromosome, while 352, named Vf0 (73 markers in chr. I, 64 in chr. II, 66 in chr. III, 74 in chr. IV, 14 in chr. V and 61 in chr. VI), were unassigned (**Supplementary Table S2**). The total map distance was 2,963.87 cM, with an average marker distance of 1.77 cM (**Table 1**). The number of markers in each linkage group (LG) ranged from 486 in LG I to 22 in LG II-1, the highest and lowest genomic distances (2.38 and 1.27 cM) were found in LGs V-2 and IV, respectively, and the maximum and minimum gap between markers (38.06 and 9.64 cM) in LGs VI and V-1, respectively. The chi-square test revealed that 34.77% of the bin markers showed distorted segregation and the highest percentages were observed in LGs V-1, II-2, and V-2 (78.26%, 64.22%, and 62.07%, respectively) (**Table 1**).

**Table 1:** Summary of the faba bean genetic map developed in this study.

Chromosome/linkage Group	Number of markers	Number of bin markers	Bin markers fitting 1:1 ratio	Bin markers outside 1:1 ratio	Total distance (cM)	Average distance (cM/bin marker)	Maximum gap (cM)
I	486	418	363	55	863.8	2.07	34.66
II/II-1	22	18	18	–	30.26	1.68	10.39
II/II-2	461	341	122	219	575.03	1.69	20.4
III	368	269	166	103	417.2	1.55	15.69
IV	454	331	235	96	420.49	1.27	16.59
V/V-1	28	23	5	18	48.33	2.1	9.64
V/V-2	81	58	22	36	137.89	2.38	19.06
VI	394	216	161	55	470.87	2.18	38.06
**Total**	2,294	1,674	1,092	582	2,963.87	1.77	

### QTL analysis

**Figure 1:**
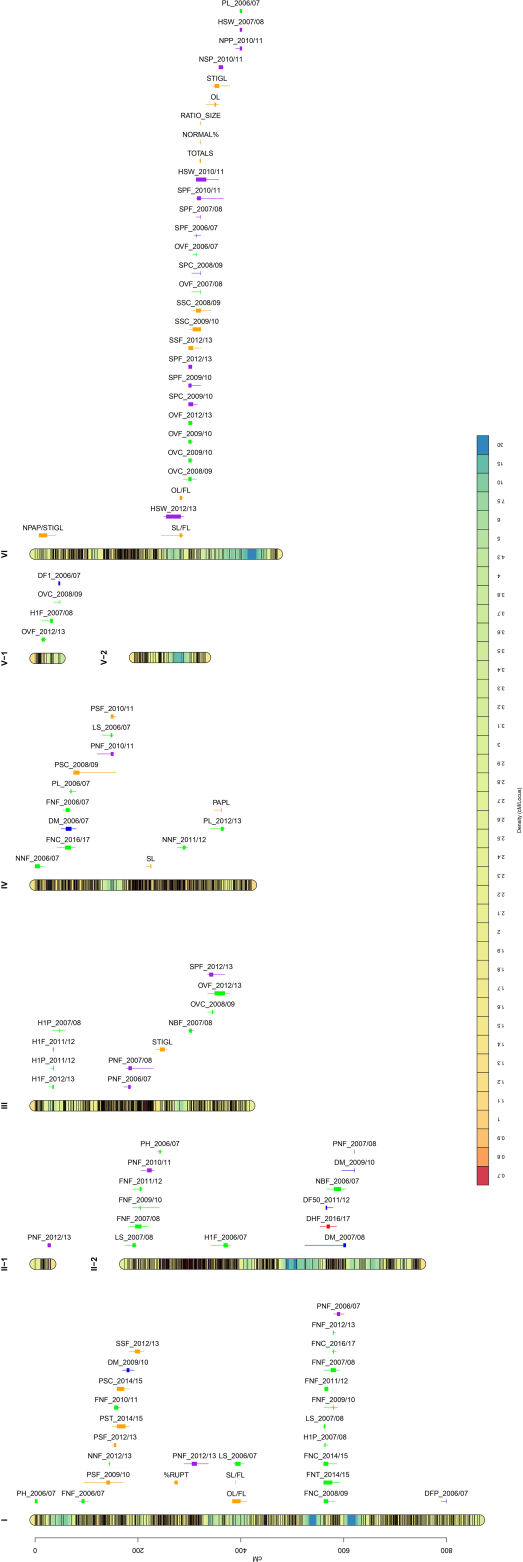
Bin marker density genetic map for the cross Vf6 x Vf27 (left side) and QTL position (right side). Color scale bar in the linkage groups ascribed to six faba bean chromosomes indicates the density of the SNP markers. Significant QTLs for the 35 traits evaluated are labeled with different colors: autofertility (orange), flowering time (blue), plant architecture (green), yield (purple), and dehiscence (red). QTL locations are represented by bars (2-LOD interval) and boxes (1-LOD interval).

The QTL analysis of 66 faba bean traits detected 99 significant QTLs corresponding to 35 traits (24 QTLs for autofertility traits, 6 for flowering time, 46 for plant architecture, 22 for yield-related traits, and 1 for dehiscence). The high-density bin map and the location of each QTL are shown in **Figure 1**. Chromosome I included 27 QTLs and chr. II 14 QTLs (one in LG II-1 and 13 in LG II-2). Furthermore, 11 QTLs were located in chr. III, 13 in chr. IV, and four in chr. V, whereas chr. VI showed 30 QTLs.

#### Autofertility

Significant QTLs for autofertility traits were detected across the whole genome except in chrs. II and V (**Figure 1**; **Table 2**), with the highest number of QTLs (11) in chr. VI. LOD scores ranged from 3.1 (NPAP/STIGL) to 8.12 (NORMAL%), with a percentage of phenotypic variation explained varying from 11.49% (NPAP/STIGL) to 27.36% (NORMAL%). Interestingly, traits related with pollen measures revealed one main zone of colocalized QTLs in chr. VI associated with the marker AX-181158608. Furthermore, three different measures for seed set (SS) were found in the same position or close to it (SSC_2008/09, SSF_2012/13 and SSC_2009/10). We found a relevant genomic region in chr. I for different pod set (PS) evaluations. Some significant QTLs for morphological floral measures were detected such as stigma length (STIGL) in chr. III and VI, style length (SL) in chr. IV, ovary length (OL) in chr. VI and their standardization with flower length (SL/FL and OL/FL) in chrs. I and VI. Furthermore, the number of stigmatic papillae divided by stigma length (N_PAP/STIGL) in chr. VI or papilla length (PAPL) in chr. IV were also detected (**Table 2**). Finally, one significant QTL related to the rupture of the stigmatic cuticle (%RUPT) was found in chr. I was associated with the marker AX-416819105 with 13.38% of variation explained. In addition, we detected some non-significant QTLs for autofertility traits that colocalized with other significant QTLs, one of which (RUPTL) was located in chr. I in the same position as %RUPT (**Supplementary Table S3**). Furthermore, a QTL for the quantity of pollen with normal size (NORMALS) located in chr. I was associated with a significant QTL of PS. Finally, a non-significant QTL for %RUPTAREA was found at a distance of 3.55 cM from the LOD peak of NPAP/STIGL QTL in chr. VI (**Supplementary Table S2**).

**Table 2:** QTLs for autofertility traits.

Chr/LG	Trait	Flanking markers (LOD -2)	Distance (cM)	LODmax	Associated marker	Position (cM)	R^2^ (%)
I	PSF_2009/10	AX-416745831-AX-181482638	74.78	3.38	AX-416737650	140.39	12.46
I	PSF_2012/13	AX-416760160-AX-181490335	6.57	5.41	AX-416796312	153.97	19.18
I	PSC_2014/15	AX-181169507-AX-416746305	23.67	3.24	AX-181494767	165.30	11.97
I	PST_2014/15	AX-416760160-AX-416746305	32.2	3.24	AX-181482638	170.6	11.97
I	SSF_2012/13	AX-416746305-AX-416751498	28.1	3.22	AX-181455524	198.74	11.90
I	%RUPT	AX-416761091-AX-416814965	7.84	3.65	AX-416819105	275.96	13.38
I	OL/FL	AX-181188351-AX-416771701	28.3	3.39	AX-416748009	385.2	12.49
I	SL/FL	AX-181487040	0	4.74	AX-181487040	389.5	17.02
III	STIGL	Vf_Mt1g095140_001-AX-416770676	20.6	6.98	Vf_Mt1g101840_001	248.01	24.02
IV	PSC_2008/09	AX-416818743-AX-181165553	55.93	3.61	AX-416752208	83.53	13.25
IV	PSF_2010/11	AX-416743756-AX-416824931	10.16	3.81	AX-416743756	147.5	13.93
IV	SL	AX-416762639-AX-416722906	10.21	3.50	AX-416726579	225.0	12.87
IV	PAPL	AX-416737303-AX-416801956	15.24	4.40	AX-416801956	362.3	15.9
VI	NPAP/STIGL	AX-416763343-AX-181179733	31.56	3.10	AX-416763343	7.83	11.49
VI	OL/FL	AX-416780927-AX-181482764	7.07	4.32	AX-416748918	283.0	15.64
VI	SL/FL	AX-181169542-AX-181482764	43.34	3.30	AX-416748918	283.0	12.18
VI	SSF_2012/13	AX-181494463-AX-181158608	23.45	4.31	AX-416810279	303.86	15.60
VI	SSC_2009/10	AX-416725236-AX-181158608	21.98	4.62	AX-416808142	309.75	16.63
VI	NORMAL%	AX-416729193-AX-181158608	1.48	8.12	AX-181158608	321.93	27.36
VI	RATIO_PSIZE	AX-416729193-AX-181158608	1.48	7.92	AX-181158608	321.93	26.78
VI	TOTALS	AX-416729193-AX-181158608	1.48	7.68	AX-181158608	321.93	26.09
VI	SSC_2008/09	AX-416763844-AX-181451152	37.86	3.54	AX-181158608	321.93	13.01
VI	OL	AX-181176378-AX-181174364	25	3.67	AX-416738786	351.4	13.45
VI	STIGL	AX-181451152-AX-416738824	36.1	3.52	AX-181174364	357.43	12.94

PSF, pod set measured in the field; PSC, pod set measured in insect proof cages; PST, pod set after tripping treatment; SSF, seed set measured in the field; %RUPT, percentage of rupture of stigma surface; OL/FL, ovary length divided by flower length; SL/FL, style length divided by flower length; STIGL, stigma length; SL, style length; PAPL, papilla length; NPAP/STIGL, number of papillae divided by stigma length; SSC, seed set measured in insect proof cages; NORMAL%, percentage of pollen normal; RATIO_PSIZE, ratio of pollen size (NORMALS/TOTALS); TOTALS, mean size of pollen grains; OL, ovary length.

#### Flowering time

We detected six QTLs for flowering time distributed in chromosomes I, II, IV, and V (**Figure 1**; **Table 3**). The QTLs explained a percentage of phenotypic variation ranging from 12.22% (DM_2009/10) to 15.2% (DF1_2006/07). QTLs for days to maturity (DM) were located in chrs. I, II, and IV: a QTL for the number of days until the 50% of the plants had open flowers (DF50) in chr. II and another QTL for the number of days to the first flower appearance (DF1) in chr. V. Despite not reaching statistical significance, a putative QTL for DM colocalized with the significant QTL for DM in chr. II and several suggestive QTLs for other flowering time measures overlapped in the DF1 QTL region in chr. V (**Supplementary Table S3**).

**Table 3:** QTLs for flowering time.

Chr/LG	Trait	Flanking markers (LOD -2)	Distance (cM)	LODmax	Associated marker	Position (cM)	R^2^ (%)
I	DM_2009/10	AX-416761809-AX-181458325	23.2	3.34	AX-416746305	183.0	12.32
II-2	DF50_2011/12	AX-416740058-AX-181492201	13.95	3.60	AX-416740058	390.9	13.21
II-2	DM_2007/08	Vf_Mt3g101420_001-AX-181489711	79.35	3.46	AX-181489711	429.7	12.73
II-2	DM_2009/10	AX-416822166-AX-181481984	24.64	3.31	AX-416822166	446.49	12.22
IV	DM_2006/07	AX-181487564-AX-181484564	29.36	3.95	AX-416798530	70.39	14.40
V-1	DF1_2006/07	AX-181488993-AX-181454097	2.64	4.19	AX-181454097	48.33	15.20

DM, days to maturity; DF50, number of days until 50% of the plants had open flowers; DF1, days from the sowing until the appearance of the first flower.

#### Plant architecture

Up to 46 significant QTLs across the whole genome were found for traits related to plant architecture (**Figure 1**; **Table 4**), with the highest number of QTLs in chromosome I (15 QTLs). LOD values ranged from 3.18 (PL_2012/13) to 8.74 (OVF_2006/07), with the percentage of phenotypic variation ranging from 11.76% to 29.11%, respectively. In total, 29 QTLs contributed over 15% of the phenotypic variation. Eight out of ten QTLs for the number of flowers per node (FN) were located in a narrow interval in chr. I, QTLs for ovules per ovary (OV) were mostly localized in chr. VI, and two of them were associated with the same marker (AX-416759442). Although the data collection was performed in different agronomic seasons, most of the QTLs colocalized or were found at very close positions, indicating that both traits are well conserved across the years. Some QTLs for the height of the first flower (H1F) and the height of the first pod (H1P) were located in chr. III, and three of them were associated with the same marker (AX-416801028). QTLs for other traits were distributed across the genome, with no specific location or aggregation pattern.

**Table 4:** QTLs for plant architecture and flower-related traits.

Chr/LG	Trait	Flanking markers (LOD -2)	Distance (cM)	LODmax	Associated marker	Position (cM)	R^2^ (%)
I	PH_2006/07	AX-181489261-AX-181489166	4.37	3.63	AX-181186095	1.46	13.31
I	FNF_2006/07	AX-416793683-AX-416724609	18.76	6.00	AX-416789822	91.3	21.03
I	NNF_2012/13	AX-181440809-AX-181444311	2.41	4.81	AX-416723849	144.8	17.25
I	FNF_2010/11	AX-416796312-AX-181494767	11.33	4.78	AX-181169507	159.37	17.15
I	LS_2006/07	AX-416748009-AX-416766054	21.35	3.32	AX-181487040	389.47	12.25
I	FNC_2008/09	AX-416739207-AX-181486180	21.28	4.58	AX-416775081	563.08	16.50
I	H1P_2007/08	AX-416739207-AX-181186015	8.34	3.78	AX-416775081	563.08	13.82
I	LS_2007/08	AX-416739207-AX-181194753	2.94	4.01	AX-416775081	563.08	14.50
I	FNC_2014/15	AX-416739207-AX-181461208	26.11	4.21	AX-181186015	569.5	15.27
I	FNF_2011/12	AX-181199624-AX-181186015	7.86	6.88	AX-181186015	569.5	23.72
I	FNT_2014/15	AX-416739207-AX-416818833	31.5	3.52	AX-181186015	569.5	12.94
I	FNC_2016/17	AX-181488566-AX-181487850	6.94	4.60	AX-416764938	579.38	16.56
I	FNF_2009/10	AX-181199624-AX-181485901	26.61	4.69	AX-181488860	579.9	16.86
I	FNF_2012/13	AX-416764938-AX-181487850	4.94	7.97	AX-416725964	580.4	26.93
I	FNF_2007/08	AX-181199624-AX-416818833	31.02	4.52	AX-181462880	583.38	16.30
II-2	LS_2007/08	AX-416753077-AX-416787674	22.96	4.39	AX-416773428	19.08	15.87
II-2	FNF_2009/10	AX-416773874-AX-416742901	51.48	4.13	AX-416824296	29.5	15.00
II-2	FNF_2011/12	AX-416773874-AX-416753159	21.19	4.09	AX-416824296	29.5	14.87
II-2	FNF_2007/08	AX-181486687-AX-416747806	37.91	3.24	AX-181494967	30.47	11.97
II-2	PH_2006/07	AX-416726884-AX-416785232	9.49	4.39	AX-181167036	68.63	15.87
II-2	H1F_2006/07	AX-416823780-AX-181491271	37.49	3.87	AX-181488476	192.41	14.13
II-2	NBF_2006/07	AX-416755553-AX-181489711	37.3	3.94	AX-416775267	410.2	14.37
III	H1F_2012/13	AX-181188340-AX-416801028	9.4	5.23	AX-416801028	36.1	18.60
III	H1F_2011/12	AX-181198441-AX-416801028	1.98	7.37	AX-416801028	36.14	25.18
III	H1P_2011/12	AX-416732900-AX-416801028	5.75	6.31	AX-416801028	36.14	21.99
III	H1P_2007/08	AX-181198441-AX-416807185	24.28	5.92	AX-181487077	47.16	20.79
III	NBF_2007/08	AX-416745204-AX-416722274	12.05	4.44	AX-416757158	299.92	16.03
III	OVC_2008/09	AX-416789004-AX-416742735	13.56	5.22	AX-416789916	344.4	18.57
III	OVF_2012/13	AX-416789004-AX-416759631	40.61	3.94	AX-416765789	351.7	14.37
IV	NNF_2006/07	AX-181472704-AX-181484108	17.49	3.59	AX-181482918	8.81	13.18
IV	FNF_2006/07	AX-181491933-AX-416798530	15.63	4.60	AX-416747764	62.0	16.56
IV	FNC_2016/17	AX-181494082-AX-416724664	34.45	4.55	AX-416774006	66.22	16.40
IV	PL_2006/07	AX-416774006-AX-181484564	13.56	3.53	AX-416817287	69.0	12.97
IV	LS_2006/07	AX-181165553-AX-416764763	21.29	5.35	AX-89898623	149.63	18.99
IV	NNF_2011/12	AX-181455568-AX-181494397	19.86	3.58	AX-181471738	289.94	13.14
IV	PL_2012/13	AX-181205360-Vf_Mt4g132020_001	28.02	3.18	Vf_Mt4g132300_001	366.3	11.76
V-1	OVF_2012/13	AX-181204556-AX-416735514	10.8	4.59	AX-416756147	15.9	16.53
V-1	H1F_2007/08	AX-181162692-AX-181189600	22.73	3.44	AX-181447376	31.6	12.66
V-1	OVC_2008/09	AX-181189600-AX-181454097	12.28	3.53	AX-181454097	48.3	12.97
VI	OVC_2008/09	AX-181482764-AX-416775308	24.78	4.71	AX-416815191	300.9	16.92
VI	OVC_2009/10	AX-181494463-AX-416763244	8.31	4.65	AX-416759442	302.4	16.73
VI	OVF_2009/10	AX-181494463-AX-416763244	8.31	6.78	AX-416759442	302.4	23.42
VI	OVF_2012/13	AX-181494463-AX-416763244	8.31	4.70	AX-416800706	304.8	16.89
VI	OVF_2006/07	AX-416763244-AX-416802051	8.53	8.74	AX-416775308	313.36	29.11
VI	OVF_2007/08	AX-416800706-AX-181158608	17.12	6.26	AX-181158608	321.9	21.84
VI	PL_2006/07	AX-416722030-AX-416822208	3.47	3.35	AX-416822208	401.83	12.35

PH, plant height; FNF, flowers per node measured in the field; NNF, number of nodes with flower; LS, leaf size; H1P, height from the base of the plant to the first pod; FNC, flowers per node measured in insect proof cages; FNT, flowers per node in the tripping treatment; H1F, height from the base of the plant to the first flower; NBF, number of branches with flowers; OVC, ovules per ovary measured in insect proof cages; OVF, ovules per ovary measured in the field; PL, pod length.

#### Yield

In total, 22 significant QTLs for yield-related traits were distributed along the faba bean genome except in chromosome V (**Figure 1**; **Table 5**). LOD values ranged from 2.46 (HSW_2010/11) to 10.65 (SPF_2006/07), with the percentage of phenotypic variation explained varying from 9.23% to 34.24%, respectively. Eight QTLs for pods per node (PN) were found in chrs. I, II, III, and IV. In chr. VI, we detected a hotspot region bearing seven QTLs for seeds per pod (SP) with a percentage of phenotypic variation explained > 14%.

**Table 5:** QTLs for yield-related traits.

Chr/LG	Trait	Flanking markers (LOD -2)	Distance (cM)	LODmax	Associated marker	Position (cM)	R^2^ (%)
I	PNF_2012/13	AX-181439520-AX-181483939	47.55	3.67	AX-416815062	313.1	13.45
I	PNF_2006/07	AX-416725964-AX-416815488	20.03	3.32	AX-181200664	591.2	12.25
I	DFP_2006/07	AX-181498191-AX-416820412	12.27	3.37	AX-181500128	799.5	12.42
II-1	PNF_2012/13	AX-181170944-AX-181490764	5.52	3.64	AX-181487044	26.7	13.35
II-2	PNF_2010/11	AX-181192959-AX-416798485	25.95	3.42	AX-416763006	47.5	12.59
II-2	PNF_2007/08	AX-416822166	–	4.67	AX-416822166	446.49	16.79
III	PNF_2006/07	AX-416792127-AX-416746489	15.56	4.18	AX-416777477	182.4	15.17
III	PNF_2007/08	AX-416803004-AX-416749998	53.04	3.30	AX-181160292	186.76	12.18
III	SPF_2012/13	AX-416789004-AX-416741311	32.92	3.56	AX-416789916	344.4	13.07
IV	PNF_2010/11	AX-181454574-AX-416764763	31.23	3.26	AX-416743756	147.5	12.04
VI	HSW_2012/13	AX-416777181-AX-181482764	38.22	3.35	AX-416780927	281.51	12.35
VI	SPC_2009/10	AX-181494463-AX-416802051	16.84	6.60	AX-416759442	302.4	22.88
VI	SPF_2009/10	AX-181494463-AX-181158608	23.45	5.55	AX-416759442	302.4	19.62
VI	SPF_2012/13	AX-181494463-AX-416800706	6.33	6.91	AX-416810279	303.9	23.81
VI	SPF_2006/07	AX-416808142-AX-416775308	3.61	10.65	AX-416775308	313.4	34.24
VI	SPC_2008/09	AX-416800706-AX-181158608	17.12	5.87	AX-181158608	321.9	20.63
VI	SPF_2007/08	AX-416775308-AX-181158608	8.57	6.76	AX-181158608	321.9	23.36
VI	SPF_2010/11	AX-416775308-AX-181152515	53.43	3.93	AX-181158608	321.9	14.33
VI	HSW_2010/11	AX-416775308-AX-181174364	44.07	2.46	AX-181176378	332.4	9.23
VI	NSP_2010/11	AX-181174364-AX-181152515	9.36	4.35	AX-181174364	357.4	15.74
VI	HSW_2007/08	AX-416722030-AX-416822208	3.47	3.16	AX-416722030	398.4	11.70
VI	NPP_2010/11	AX-416724490-AX-416822208	11.88	4.02	AX-416722030	398.4	14.63

PNF, number of pods per node measured in the field; DFP, days to first pod; SPF, seeds per pod measured in the field; HSW, hundred seed weight; SPC, number seeds per pod measured in insect proof cages; NSP, number of seeds per plant; NPP, number of pods per plant.

#### Dehiscence

Only one QTL was significant for the different dehiscence traits. This QTL was located in chromosome II (**Figure 1**; **Table 6**), with an LOD of 3.41, and it explained 12.56% of the phenotypic variation. Interestingly, we also found non-significant QTLs for other dehiscence traits in chrs. III, IV, and VI co-localizing with seed and pod-related traits (**Supplementary Table S3**).

**Table 6:** QTL for dehiscence.

Chr/LG	Trait	Flanking markers (LOD -2)	Distance (cM)	LODmax	Associated marker	Position (cM)	R^2^ (%)
II-2	DHF_2016/17	Vf_Mt3g100050_001-AX-181148576	32.07	3.41	AX-181486469	395.3	12.56

DHF, number of dehiscent pods measured in the field.

#### Non-significant QTLs

We observed 46 minor QTLs that did not reach the LOD threshold value for statistical significance but colocalized with other significant QTLs (**Supplementary Table S3**). In total, 16 were located in chr. I, 6 in chr. II, 5 in chr. III, 8 in chr. IV, 7 in chr. V, and 4 in chr. VI. Furthermore, 10 of these QTLs correspond to autofertility traits, 5 to flowering time traits, 9 to plant architecture traits, 17 to yield-related measures, and 5 to dehiscence. The comparison of the map position revealed that four minor QTLs associated with hundred seed weight (HSW), PNC, NLL, NSP, and NPP in chr. I overlapped in a zone bearing several significant QTLs for pod set (PS) and FN. A similar situation was found in chr. IV, where a group of non-significant QTLs colocalized with QTLs for PNF, PSF, and LS. As mentioned above, in chr. V, several non-significant flowering time-related QTLs colocalized in the region with statistically significant QTLs for DF1 and OVC.

#### Colocalizing groups

Across the linkage map, we identified 13 zones with at least three colocalizing QTLs (**Figure 1**; **Supplementary Table S4**). Three of them were found in chr. I and VI; two in chrs. II, III, and IV; and one in chr. V. We numbered the groups following the order of appearance (1-13). Group 1 included five QTLs, three of them belonging to pod set traits in chr. I. Group 3 in chr. I and group 4 in chr. II gathered eight and three colocalized QTLs for flowers per node, respectively. Group 5, clustered together three flowering time QTLs. Finally, group 12 contained 23 colocalized QTLs for plant architecture, autofertility, and yield-related traits (**Supplementary Tables S2**, **S4**). Phenotypic correlations between groups of traits are shown in **Supplementary Figure S1**. Most of them showed significant positive correlations (p < 0.05), ranging from 0.20 to 0.99. By contrast, only a few significant negative correlations were found in groups 1, 5, 10, and 12, ranging from -0.21 to -0.52. In group 1, PS, FN, and DM were negatively correlated. Groups 3 and 4 revealed a high (r > 0.50) significance and positive correlations between traits belonging to plant architecture. In group 5, DH and PN displayed significant negative correlations with the flowering time traits, whereas in group 8, DM showed a significant positive correlation with different plant architecture traits. In group 10, DF1 displayed a significant negative correlation with the number of ovules per ovary. In group 12, we observed significant positive correlations between traits of the same category and between different autofertility (pollen traits), yield (SP), and plant architecture traits (OV). Finally, in group 13, HSW and PL revealed a highly significant positive correlation. For each QTL or colocalizing group, we estimated the number of genes included in the genetic intervals, according to the genome physical map (**Supplementary Table S4**). A total of 3,967 genes were found in those regions spanning 2281.42 Mbp. The number of genes in the groups of colocalized QTLs ranged from 14 (group 13) to 929 genes (group 9) and the physical distances ranged from 6.84 Mbp (group 13) to 571.24 Mbp (group 9). The rest of the groups revealed a mean distance of 140.02 Mbp with an average of 250 genes.

## Discussion

### Genetic map and QTL detection

Genomic resources in faba bean have lagged behind other grain legume species, a fact attributed to the lower investment combined with the difficulties in assembling its giant genome of ~13 Gb. In default of a reference genome, exploiting the synteny with closely related and sequenced species, such as *Medicago truncatula, Cicer arietinum*, or *Pisum sativum*, allowed researchers to develop the first gene-based genetic maps, identify quantitative trait loci, and in a few cases, determine candidate genes. Transcriptome sequencing from different tissues or conditions (Ocaña et al., 2015; Webb et al., 2016; Khan et al., 2019; Lyu et al., 2021; Casimiro-Soriguer et al., 2024) led to comprehensive transcript databases useful for saturation mapping. Present advances in next-generation sequencing platforms and high-throughput genotyping techniques are providing enormous advances in faba bean (He et al., 2014; Annicchiarico et al., 2017; Varshney et al., 2019; Gutierrez et al., 2023, 2024). Thus, recent publications (Li et al., 2023; Zhao et al., 2023) describe the development of the most ultra-dense genetic maps (with 5,103 and 12,023 markers, respectively) reported thus far in this crop, using SNP markers derived from targeted next-generation sequencing (TNGS) genotyping platforms. Here, we have developed a new high-density genetic map in the RIL population Vf6 x Vf27 consisting of 124 F_8:9_ individuals using the Vfaba_v2 Axiom 60K SNP array and we have mapped a total of 35 traits detecting 99 significant QTLs. After quality control, we obtained 2,296 SNP markers, grouped into 1,674 bins, that were used to construct the genetic map. The recent publication of the faba bean genome sequence (Jayakodi et al., 2023) allowed us to assign the LGs to chromosomes. Moreover, 352 markers formerly unassigned to a physical position (Vf0), could be ascribed to chromosomes thanks to the construction of this genetic map (**Supplementary Table S2**). Population size is a key factor determining QTL position and effects and the power of the analysis increases with larger populations (Vales et al., 2005; Alonso-Blanco et al., 2006; Wang et al., 2012). In biparental QTL mapping analysis, the standard number of lines used (approximately 100) may underestimate the number of QTLs with small effects. Nevertheless, despite the relatively limited population size used in this study, the major QTLs detected in several years are conserved as would be in a larger population (Vales et al., 2005).

The proportion of bin markers with distorted segregation considering all the chromosomes was relatively high (34.77%), with LG V-1 having the highest distortion rate (78.26%). The distorted markers did not affect the grouping in the map, a fact supported by the high LOD threshold value (10.5) used, thus suggesting that distortion may be due to biological mechanisms. Distorted segregation is common in most plant genetic mapping experiments and has been mainly associated with preferential gametic or zygotic selection affecting recombination frequency (Li et al., 2011; Dai et al., 2016; Zhang et al., 2019). Due to the fear of unpredictable consequences in the results, distorted markers have usually been excluded in linkage map construction and QTL mapping (Zhan and Xu, 2011). However, the removal of these markers results in a loss of information that decreases marker density and increases the average marker distances. Different authors have demonstrated that distorted segregation can be incorporated into existing QTL mapping studies without compromising the accuracy of the genetic maps or the QTL detection (Zuo et al., 2019). In this study, we kept the markers that showed distortion in the Mendelian segregation (> 30%) to build the genetic map and perform the QTL analysis. This could explain the larger genetic distance achieved (2,963.87 cM) compared to other recent high density faba bean maps (Li et al., 2023; Zhao et al., 2023), confirmed by the fact that the removal of distorted markers from our dataset greatly reduced the genetic map distance (data not shown).

### Genetic and physical map

The comparison of the physical and genetic maps, using the Pretzel platform (Keeble-Gagnère et al., 2019; http://pulses.plantinformatics.io/mapview), showed a good correspondence between chromosomes and linkage groups, especially in chrs. I, II, and IV (**Supplementary Figure S2**). Chromosomes II and V showed a division in two different linkage groups (1 and 2), and despite this, they showed a good correspondence with the physical map. Chromosomes III and VI showed several twists in the marker order. Furthermore, we found huge gaps in chromosomes IV and V. The gap in chr. IV was located around 150 cM, corresponding to the physical chromosome section between 300 Mbp and 1 Gbp. The gap in chr. V coincided with the division in the two linkage groups and corresponds to a distance from 400 Mbp to 1 Gbp in the physical chromosome (**Supplementary Figure S2**). We have observed that most of the markers that had been removed because of their high distortion rates are located in this gap region and could explain the division of chromosome V due to the limited number of markers in this area.

To identify candidate genes for autofertility, flowering time, plant architecture, yield, and dehiscence traits, the genomic sequences of the significant markers associated with a QTL were searched for homologs in the *M. truncatula* and *A. thaliana* genomes. In the next sections, we will discuss the QTLs detected consistently in previous studies and the putative candidate genes harboring the significant markers clearly related with the trait.

### Autofertility

Several traits related to autofertility were analyzed in this work, including pod set, seed set, and floral measurements taken on the male and female structures. The results revealed 24 QTLs for autofertility traits in all chromosomes except chrs. II and V (**Table 2**). A previous study (Aguilar-Benitez et al., 2022) reported a QTL for pollen size in chr. VI with a close position to the significant marker detected in this study [AX-181158608, (Vfaba.Hedin2.R1.6g115480.2)]. Similarly, in the same study, we also found two QTLs for seed set (number of seeds/number of ovules x 100) in chr. VI associated with Mtr4g088524, which is closely positioned to the significant markers detected in this study for seed set in the field or in insect-proof cages (**Supplementary Table S5**).

Two markers associated with the seed set QTL detected in this study showed functions related to pollen germination and pollen tube growth. The first one was AX-181455524 (Vfaba.Hedin2.R1.1g093640.1) in chr. I. corresponding to a “chloride channel C” (**Supplementary Table S6**), which is expressed during pollen germination and pollen tube growth (Wang et al., 2008). The second marker, AX-181158608 (Vfaba.Hedin2.R1.6g115480.1) located in colocalizing group 12 (chr. VI) (**Supplementary Tables S2**, **S4**, **S6**), was identified as a “cellulose synthase-like G2 protein”, responsible for cellulose synthesis and important for a correct pollen tube growth (Cai et al., 2011) and essential for the pollen wall. Mutations in this enzyme showed aberrant pollen morphology and male sterility (Persson et al., 2007).

For pod set (number of pods/number of flowers x 100), three QTLs were detected in chr. I, colocalizing in group 1 with a genetic interval of 32.2 cM. The significant marker AX-181494767 associated with PSC_2014/15 showed homology with a protein kinase homolog to a “Sid1 protein” in yeast (Van Damme et al., 2004) (**Supplementary Table S6**). This protein is implicated in cytokinesis associated with the spindle poles, and it is important in cell proliferating tissues such as seeds and pods. The significant marker AX-181482638 associated with PST_2014/15 showed as ortholog a “Teosinte Branched 1 (TCP3) protein”, which is related to cytokinin signaling pathways implicated in organogenesis (Li and Zachgo, 2013; Yang et al., 2020). Furthermore, we detected two QTLs for pod set in chr. IV. The significant SNP marker (AX-416752208) linked to PSC_2008/09 (**Supplementary Table S6**) was identified as a “phosphatidylinositol-4-phosphate 5-kinase family protein (PIP5K6)”. Mutants for this gene in *Arabidopsis thaliana* showed defects in pollen germination and pollen tube growth, affecting fecundation and formation of siliques (Ischebeck et al., 2008; Kato et al., 2024).

Female morphological traits (e.g., style, ovary, or papilla morphology and number of stigmatic papillae) revealed QTLs in chrs. I, III, IV, and VI. The style length (SL) QTL detected in chr. IV in the physical interval 4g165880-4g171920 revealed a close position to the previously reported QTL (Aguilar-Benitez et al., 2022) for the same trait (**Supplementary Tables S2**, **S5**). The significant marker belongs to a bin marker composed of four markers. Two of these, AX-416790655 and AX-416745029 (**Supplementary Table S6**), corresponded to ortholog proteins whose biological functions were related to actively growing tissues, including flower development (Bessire et al., 2011; Fabre et al., 2016; Jin et al., 2019; Zhu et al., 2020). Furthermore, QTLs for OL/FL and SL/FL in colocalizing group 11 in chr. VI (**Supplementary Table S4**) showed associated markers related with an “ethylene responsive element binding factor 2 (ERF2)” implicated in ethylene signaling in *Arabidopsis* (**Supplementary Table S6**). This hormone in cucurbits affects ovary length during flower development (Boualem et al., 2022).

### Flowering time

We detected six QTLs for flowering time in chrs. I, II, IV, and V, as previously reported by Cruz-Izquierdo et al. (2012); Catt et al. (2017), and Aguilar-Benitez et al. (2021). Nevertheless, the position of the QTL for DF1 in chr. V identified by Aguilar-Benitez et al. (2021) was not conserved in this work, probably due to the removal of the most distorted markers, including some of the markers closely located to flowering time genes (FT).

A QTL for DF50 in chr. II detected in this study showed a close position to that previously described by Aguilar-Benitez et al. (2021) (**Supplementary Table S5**).

Two QTLs for DM were located in chr. II (**Supplementary Table S6**), and one of the significant SNP markers (AX-181489711) was identified as a “phosphatidic acid phosphohydrolase 2 protein” implicated in the regulation of glycolipids, signaling molecules involved in flower development (Nakamura et al., 2014). The other significant marker AX-416822166 corresponds to a “casein kinase I-like 3”, which regulates the activity of cryptochromes by blue light (Tan et al., 2013). Both genes could affect flowering time and pod development in faba bean (Kang and Wang, 2020).

### Plant architecture

This group of traits is the most extensive, including traits for different parts of the plant (e.g., flowers, leaves, and stems). In this study, we detected 46 QTLs for architectural traits along the genome with interesting and conserved regions for some specific traits. The trait FN was highly conserved between evaluations. Several QTLs colocalized in groups 3, 4 and 8, being most of them in chr. I in a range of ~32 cM (**Table 4**; **Figure 1**; **Supplementary Tables S2**, **S4**). The significant SNP markers (AX-181191222, AX-181490810, AX-416758150) associated to QTLs for FN in chr.I showed a close position with the previously QTL reported by Avila et al. (2017). The significant marker AX-181488860 identified for FNF_2009/10 in chr.I (**Supplementary Table S6**), the candidate gene, was a “FG-GAP repeat-containing protein” (*NERD1*) in *Arabidopsis*, implicated in several aspects of plant growth and development, including root and shoot growth and flowering. This gene has been related with changes in the number of flowers in *Arabidopsis* (Yuan and Kessler, 2019; Xu et al., 2020), as could be the case for faba bean.

Interestingly, a recent GWAS analysis identified two marker-trait associations (MTAs) for plant height (PH) in chr. I (Skovbjerg et al., 2023; Ohm et al., 2024). We checked the location of these MTAs in our QTLs and found that AX-416796020 is the same significant marker described by Skovbjerg et al. (2023), and D15935 was close to the region defined by the flanking markers for PH_2006/07 in chr.I, thus supporting our results (**Supplementary Table S5**). Moreover, MTAs previously identified for pods per plant (PP), seed per plant (SPL), and HSW (Gutierrez et al., 2023, 2024) showed close physical positions to QTLs for NNF and PL in chr. IV described in the current study (**Supplementary Table S5**). Marker AX-416788562 for PP and SPL identified by Gutierrez et al. (2024) was close to the NNF_2011/12 QTL, indicating a possible relationship between the number of nodes with flowers and yield in faba bean genotypes. In the case of HSW, the associated marker AX-416740666 reported by Gutierrez et al. (2023) was now next to PL_2012/13, pointing to a possible relationship between pod length and seed weight. In a recent work, Li et al. (2023) developed high-density maps in two faba bean populations and identified several QTLs for plant architectural traits. The locations of QTLs for PH (chrs. I and II), H1F (chrs. II, III and V), and H1P (chrs. I and III) were consistent with the results presented here.

The five QTLs detected for H1F and H1P in chrs. I and III showed close physical positions to the previously described by Avila et al. (2017) (**Supplementary Table S5**). The significant marker AX-181488476, associated with H1F in chr. II, corresponds to a “galacturonosyltransferase-like 3 (GATL3) protein” (**Supplementary Table S6**). In *A. thaliana*, a mutant in a gene of the same family showed a reduction of inflorescence stem height of 20% in comparison with wild-type (Lao et al., 2003). Similarly, the AX-416801028 marker associated with the H1P and H1F colocalizing group 6 (**Supplementary Tables S4**, **S6**) was identified as a “SMAX1-like 3 protein” in *Arabidopsis*. Mutations in a member of this gene family showed a reduced primary inflorescence height in *Arabidopsis* plants (Stirnberg et al., 2002).

The significant marker AX-89898623, associated with the LS QTL in chr. IV, (**Supplementary Table S6**) was identified as a “SUMO-conjugating enzyme (SCE1)”. SUMO family proteins are responsible for posttranscriptional modifications regulating transcription factor activity, subcellular location, and protein-protein interactions. A SUMO protein of *A. thaliana* showed a relationship with the leaf elongation and enlargement, affecting the total leaf area (Catala et al., 2007).

OV was a very stable trait belonging to colocalizing groups 7, 10, and 12 and ascribed to chrs. III, V, and VI (**Table 4**; **Figure 1**; **Supplementary Table S4**). AX-416775308, associated with OVF_2006/07 in the colocalizing group 12, was identified as an “Auxin Response Transcription Factor 3 (ARF3)” (**Supplementary Table S6**) previously implicated in carpel and ovule development (Sessions et al., 1997; Kelley et al., 2012; Andres-Robin et al., 2018).

### Yield

In total, QTLs for yield-related traits were distributed across the genome (except chr. V), although the major genomic region was detected in chr. VI (**Table 5**, **Supplementary Tables S2**, **S4**). Seven QTLs for SP were identified in the colocalizing group 12 (chr. VI) and the significant markers were close to QTLs previously detected for this trait (Cruz-Izquierdo et al., 2012; Avila et al., 2017) (**Supplementary Table S5**). Similarly, significant markers for the QTL for HSW reported by Avila et al. (2017) showed a close position with a QTL detected in this study (HSW_2012/13) in chr. VI (**Supplementary Table S5**). Our results were also supported by Li et al. (2023) who identified QTLs for HSW in chr. VI.

Furthermore, AX-181205104, the significant marker for PNF_2007/08 detected in chr. III in this study, was the same as that reported in a recent GWAS analysis (Gutierrez et al., 2024) for SPL and shattering (SH), showing a possible relationship between these yield-related traits.

Several proteins with a wide variety of functions were identified by the associated markers but only few of them showed biological functions that were apparently related with these traits. Thus, two markers associated to pods per node in chrs. I (AX-416815062) and II (AX-181487044), (**Supplementary Table S6**) were involved in the distribution of siliques in *A. thaliana* (Rodor et al., 2011; Yang et al., 2012). Compared with the wild-type, mutants in these genes showed variations in the spatial position of siliques or even a total lack of siliques.

A marker associated with the QTL for the number of days to the first pod (DFP) in chr. I (AX-181500128) was orthologous to an *Arabidopsis* gene whose function was related to seed coat development, specifically the cellulosic microfibrils (Yang et al., 2022). This outcome points towards a possible relationship between the timing of pod formation and the correct development of the seed’s coat.

The significant marker AX-416759442, associated with QTLs for OV and SP, located in group 12 (chr. VI) and highly correlated between them (**Supplementary Tables S2**, **S4**, **S6**; **Supplementary Figure S1**), was identified as a transmembrane protein implicated in pollen germination and pollen tube growth, affecting fertilization and seed and fruit formation (Lalanne et al., 2004).

### Dehiscence

Evaluation of pod dehiscence is complicated because pod shattering is highly affected by environmental conditions. Several studies evaluating the dehiscence in different species have identified a variety of genes implicated in cell wall formation and composition (Funatsuki et al., 2014; Ballester and Ferrándiz, 2017; Aguilar-Benitez et al., 2020a, b; Di Vittori et al., 2021). In this study, a QTL in chr. II was identified close to a previously QTL reported by Aguilar-Benitez et al. (2020a) (**Supplementary Table S5**). The significant marker associated, AX-416755553 (**Supplementary Table S6**), was identified in *Arabidopsis* as a “CLPC homologue 1 protein”, localized into chloroplast and related to chloroplast development and degradation. Mutants in this gene showed a reduced photosynthetic capability and evident chlorosis (Sjögren et al., 2004). Paralog genes showed a high expression during different abiotic stresses, including dehydration and senescence (Nakashima et al., 1997; Simpson et al., 2003), indicating a possible relationship between senescence and pod drying and this gene.

### Colocalizing groups

The present study identified several colocalizing groups of traits linking PS or PN with FN and flowering time traits (groups 1, 3, 4, and 5, **Supplementary Table S4**). Egli (2005), studying the reproductive process in soybean, described that the pod set was affected by the position of flowers and the flowering time, indicating a high pod abortion in cultivars with late and long flowering time periods. Negative correlations obtained in this study between flowering time traits (DF and DM) and PS and PN (groups 1 and 5, **Supplementary Figure S1**) support the relationship among these traits. Similarly, the positive correlation found in this study between PL and HSW in group 13 (**Supplementary Figure S1**) is in agreement with previous studies in soybean (Bravo et al., 1980) or *Brassica napus* (Li et al., 2019), indicating that pod length and width are significantly correlated with the final seed size and can be used as an indirect selection for final yield.

The largest colocalizing group, including OV, SP, and SS in group 12 (**Figure 1**; **Supplementary Table S4**), was positively correlated (**Supplementary Figure S1**), evidence that the number of ovules per ovary determines the maximum number of seeds per pod. According to Qadir et al. (2021), the biological regulation of OV and SP implicates several plant hormones, such as auxin. Previously, we have described an auxin transport (*ARF3*) as the candidate gene for the ovules per ovary QTL in chr. VI. This hormone regulates the gynoecium and ovule development through several transporters and transcription factors. A recent work in pigeonpea (*Cajanus cajan*) showed an overexpression of *ARF3* in a line with a high number of seeds per pod in comparison with a line with a lower number of seeds (Arpita et al., 2023). Our results were consistent with these previous studies, pointing to *ARF3* as a possible candidate for OV and SP traits.

Although QTL mapping in biparental populations provides high statistical power for detecting a QTL, the resolution is still low due to the limited genetic diversity existing between the parents. A GWAS is an additional tool to dissect polygenic traits by utilizing the genetic diversity and historical recombination events present in wide germplasm collections. An association analysis increases the mapping resolution and enables minor effect genes to be detected. The combination of both methods may help identify the causal gene or mutation and provide new insights into the genetic architecture of complex traits.

Some of the MTAs recently reported (Gutierrez et al., 2023, 2024) fall inside the colocalizing groups described in this study. Thus, the MTA AX-416785709 (no significant similarity found), related to the number of pods per plant under drought conditions (Gutierrez et al., 2023), was found in group 5 (chr. II), including flowering time, dehiscence, plant architecture, and yield-related traits. Furthermore, the MTA AX-416807594, also related to the number of pods per plant (Gutierrez et al., 2024), was found in group 7 (chr. III), where QTLs for ovules per ovary and number of seeds per pod were described. The candidate gene corresponding to this marker was identified as an “ACTIVITY OF BC1 COMPLEX KINASE 1 protein” in *Arabidopsis* (Gutierrez et al., 2024) and its function was related to the regulation of reactive oxygen species (ROS) (Qin et al., 2020). ROS play a crucial role during female gametogenesis and fertilization in *A. thaliana* (Martin et al., 2013), thus suggesting a putative relationship among the number of ovules, seeds, and pods in faba bean. Finally, the MTA AX-416801028, associated with plot yield (Gutierrez et al., 2024), was located in group 6 (chr. III), an important genomic region with QTLs for H1P and H1F, indicating a positive relationship between the height to first inflorescence and the final yield per plot.

## Conclusions

In the present study, the genotyping data generated from a faba bean RIL population using the ‘Vfaba_v2’ SNP genotyping array has facilitated the construction of a high-density genetic linkage map including 2,296 SNP markers that cover 2,963.87 cM with an average marker distance of 1.77 cM. A comparison of the physical and genetic maps revealed a good correspondence between linkage groups and chromosomes. QTL analysis of the phenotyping data from 66 traits, previously evaluated across several years, led to the identification of 99 significant QTLs corresponding to 35 traits. A large percentage of QTLs were stable across years and environments and represent important targets for breeding, as they correspond to highly heritable genomic regions and those less affected by environmental factors. A major finding in the study was the colocalization of QTLs (including three or more overlapping QTLs) in 13 major genomic regions. The integration of the QTL mapping with recently reported GWAS data has been useful to uncover and refine the most consistent genomic regions related to several yield-related traits. After further QTL fine-mapping and candidate gene validation, exploring the functional significance of these candidates or integrating genomic data with physiological studies will enhance the understanding of trait regulation in faba bean and will provide targets for future marker assisted selection in this crop species.

## Data Availability

The original contributions presented in the study are publicly available. This data can be found here: https://doi.org/10.6084/m9.figshare.28668263.v1.
