# Supplementary material for: A high-density linkage map and fine QTL mapping of architecture, phenology, and yield-related traits in faba bean (Vicia faba L.)
Source: Front Plant Sci. 2025 Apr 7;16:1457812. doi: 10.3389/fpls.2025.1457812 (PMC12009772; doi:10.3389/fpls.2025.1457812)
Supplement: Supplementary file 1 [file DataSheet1.zip › Supplementary Figure S2.PDF]

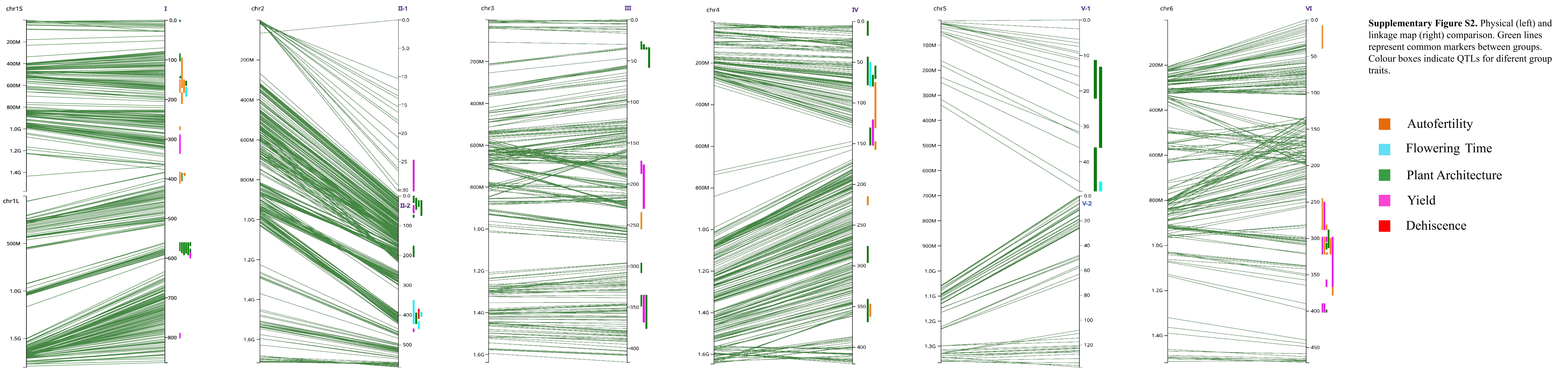

Supplementary Figure S2. Physical (left) and linkage map (right) comparison. Green lines represent common markers between groups. Colour boxes indicate QTLs for diferent group traits.
