# Supplementary material for: A high-density linkage map and fine QTL mapping of architecture, phenology, and yield-related traits in faba bean (Vicia faba L.)
Source: Front Plant Sci. 2025 Apr 7;16:1457812. doi: 10.3389/fpls.2025.1457812 (PMC12009772; doi:10.3389/fpls.2025.1457812)
Supplement: Supplementary file 1 [file DataSheet1.zip › Supplementary Figure S1.PPTX]

## Slide 1
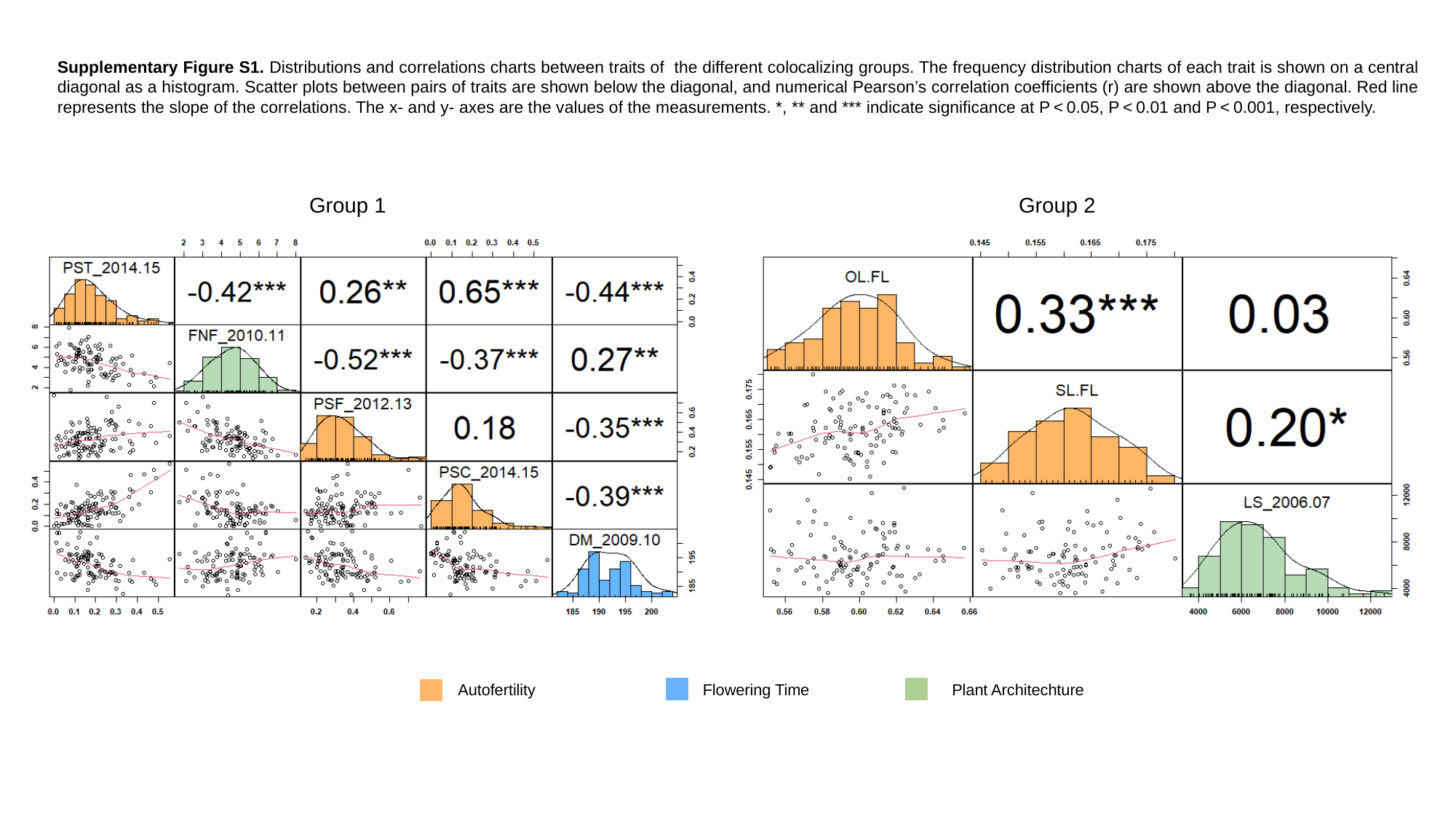

Supplementary Figure S1. Distributions and correlations charts between traits of the different colocalizing groups. The frequency distribution charts of each trait is shown on a central diagonal as a histogram. Scatter plots between pairs of traits are shown below the diagonal, and numerical Pearson’s correlation coefficients (r) are shown above the diagonal. Red line represents the slope of the correlations. The x- and y- axes are the values of the measurements. *, ** and *** indicate significance at P < 0.05, P < 0.01 and P < 0.001, respectively.
Group 2
Group 1
Autofertility
Flowering Time
Plant Architechture

## Slide 2
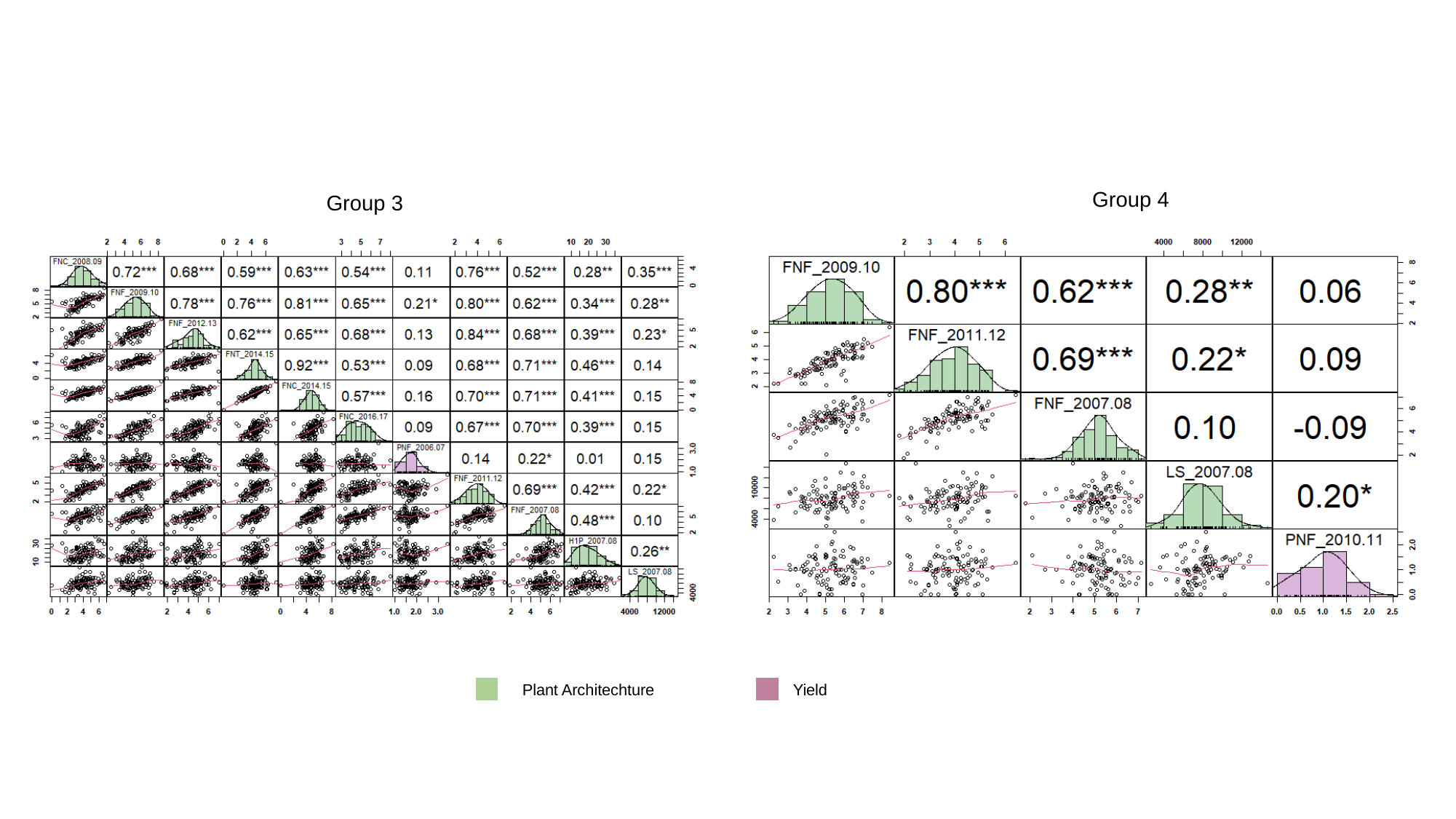

Group 4
Group 3
Plant Architechture
Yield

## Slide 3
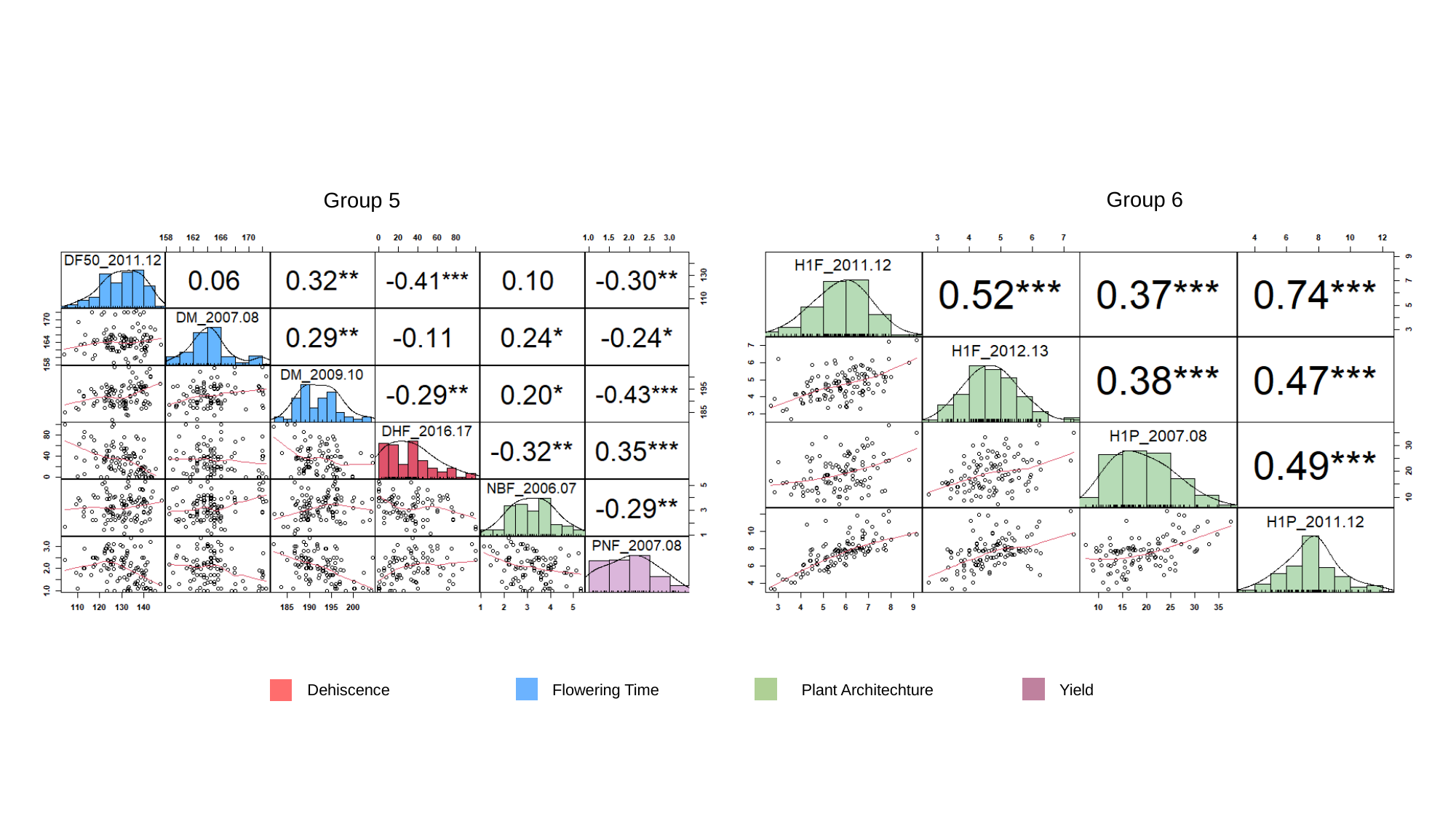

Group 6
Group 5
Dehiscence
Flowering Time
Plant Architechture
Yield

## Slide 4
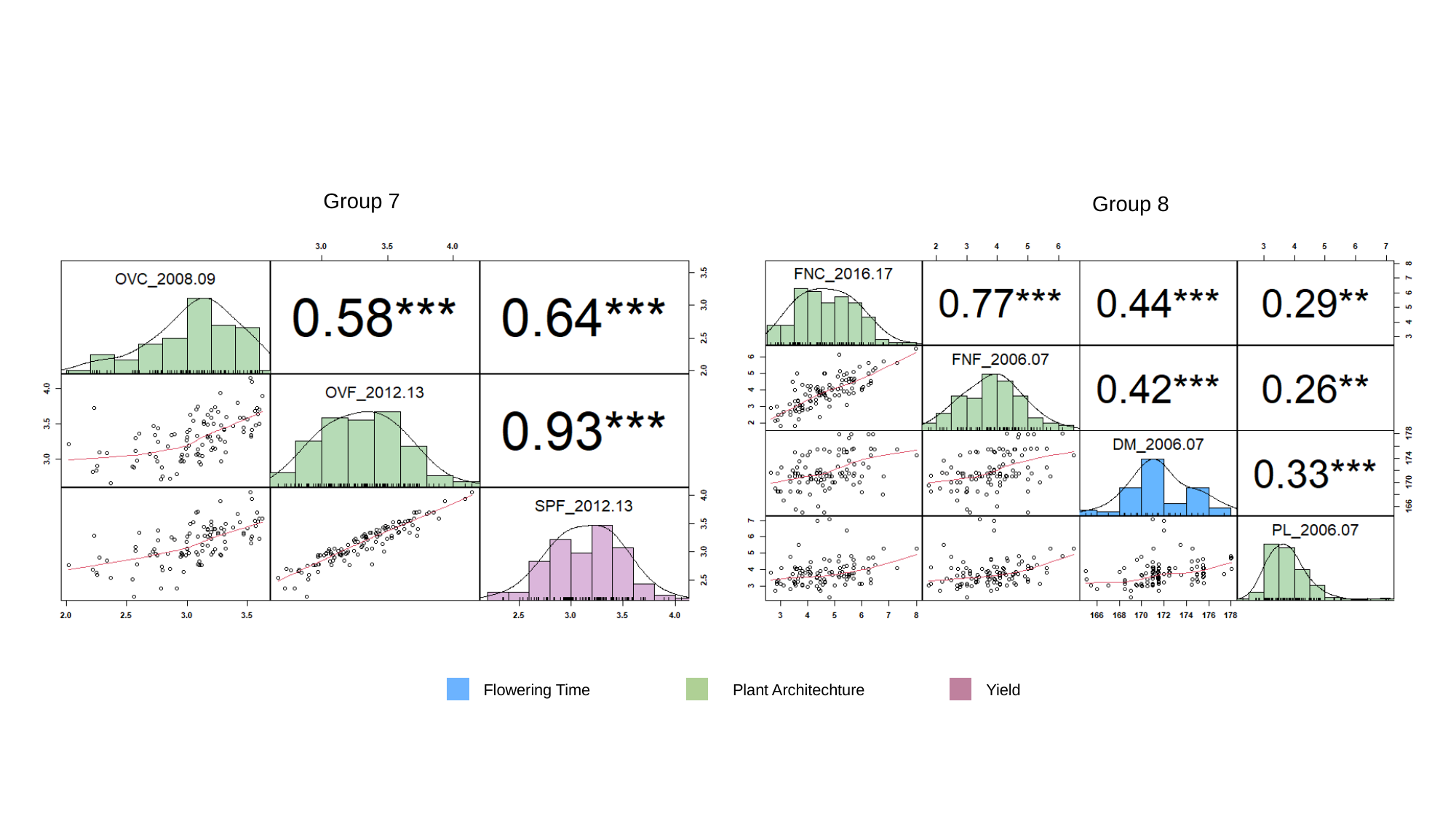

Group 7
Group 8
Flowering Time
Plant Architechture
Yield

## Slide 5
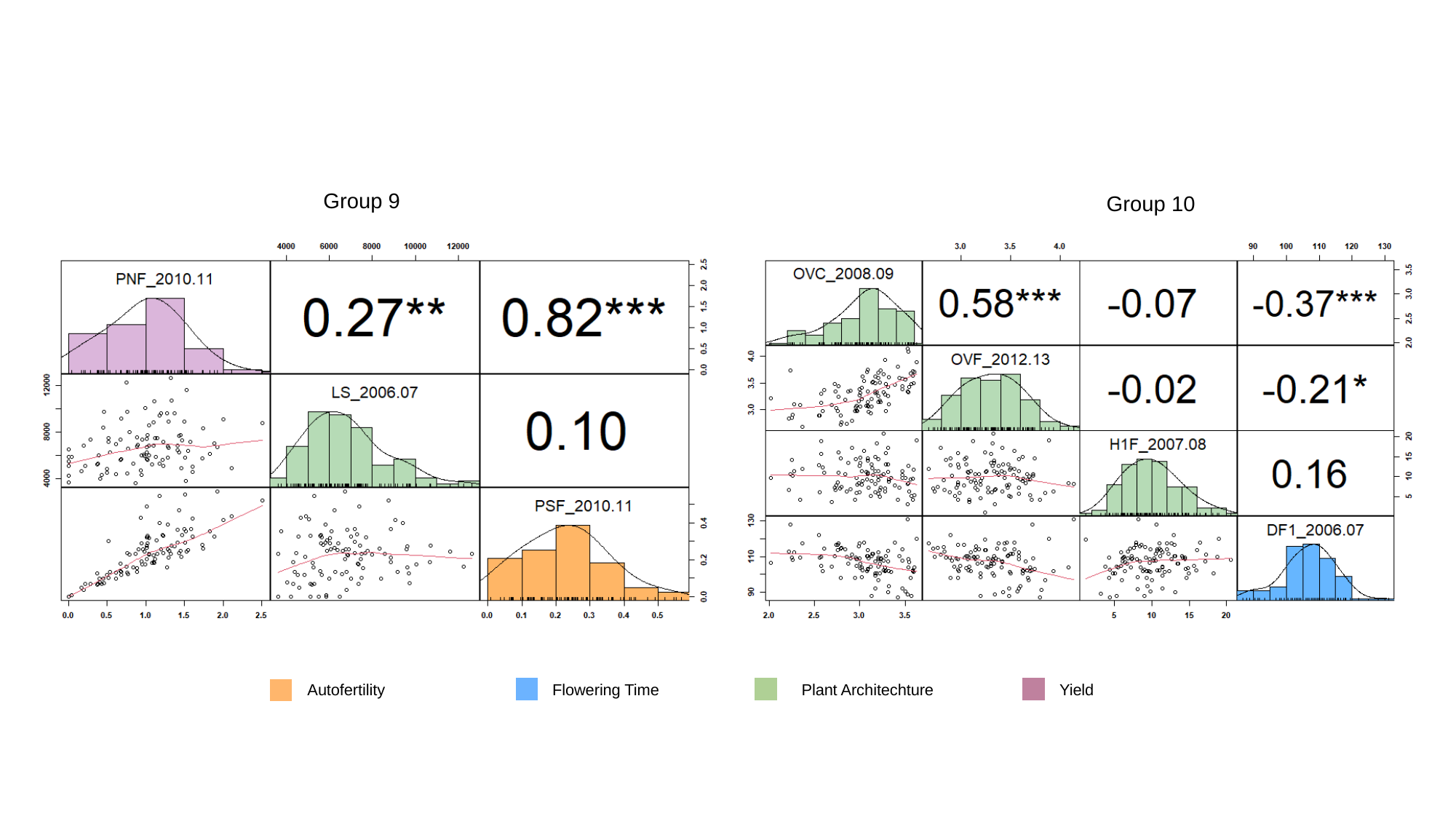

Group 9
Group 10
Autofertility
Flowering Time
Plant Architechture
Yield

## Slide 6
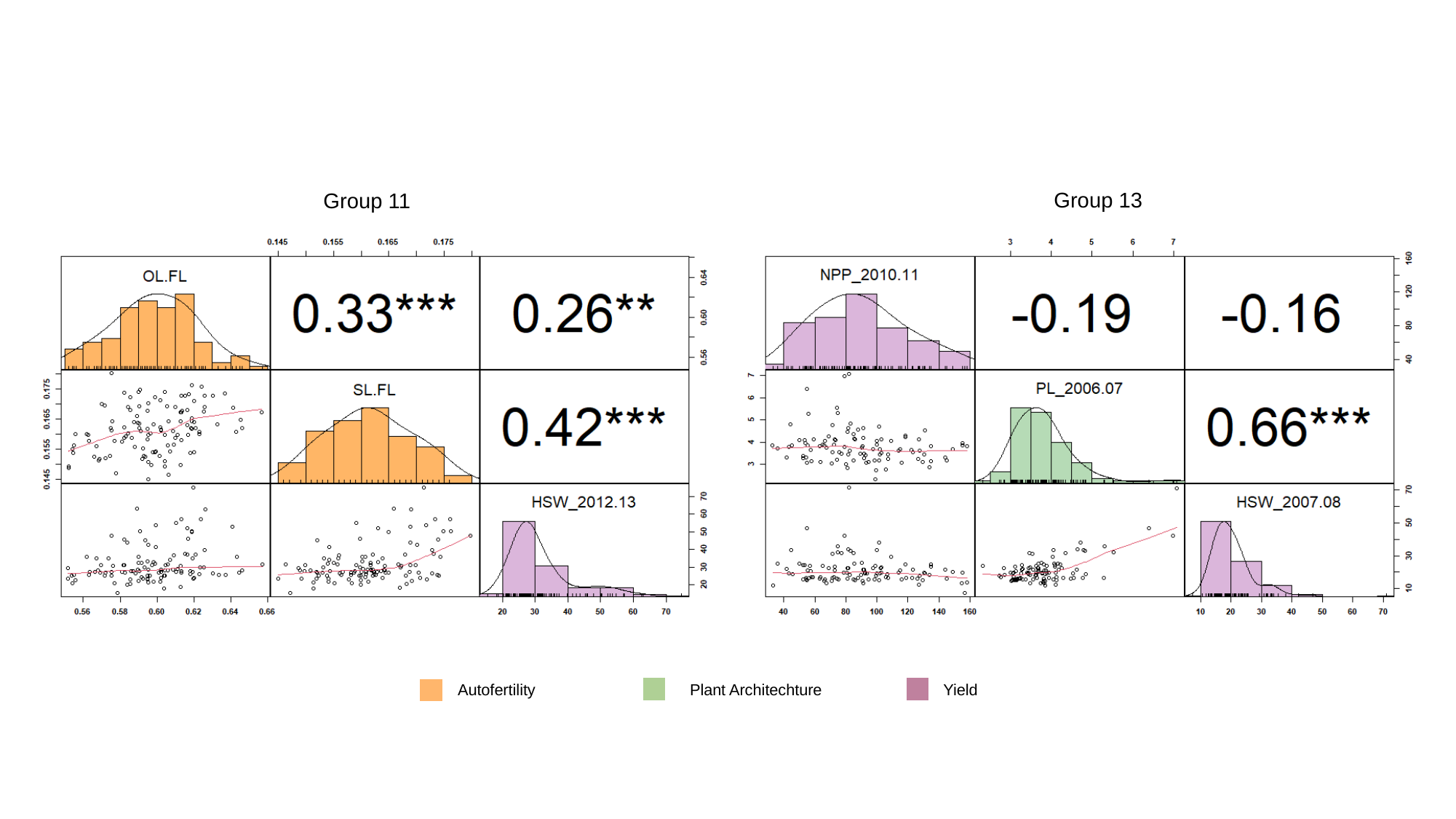

Group 13
Group 11
Autofertility
Plant Architechture
Yield

## Slide 7
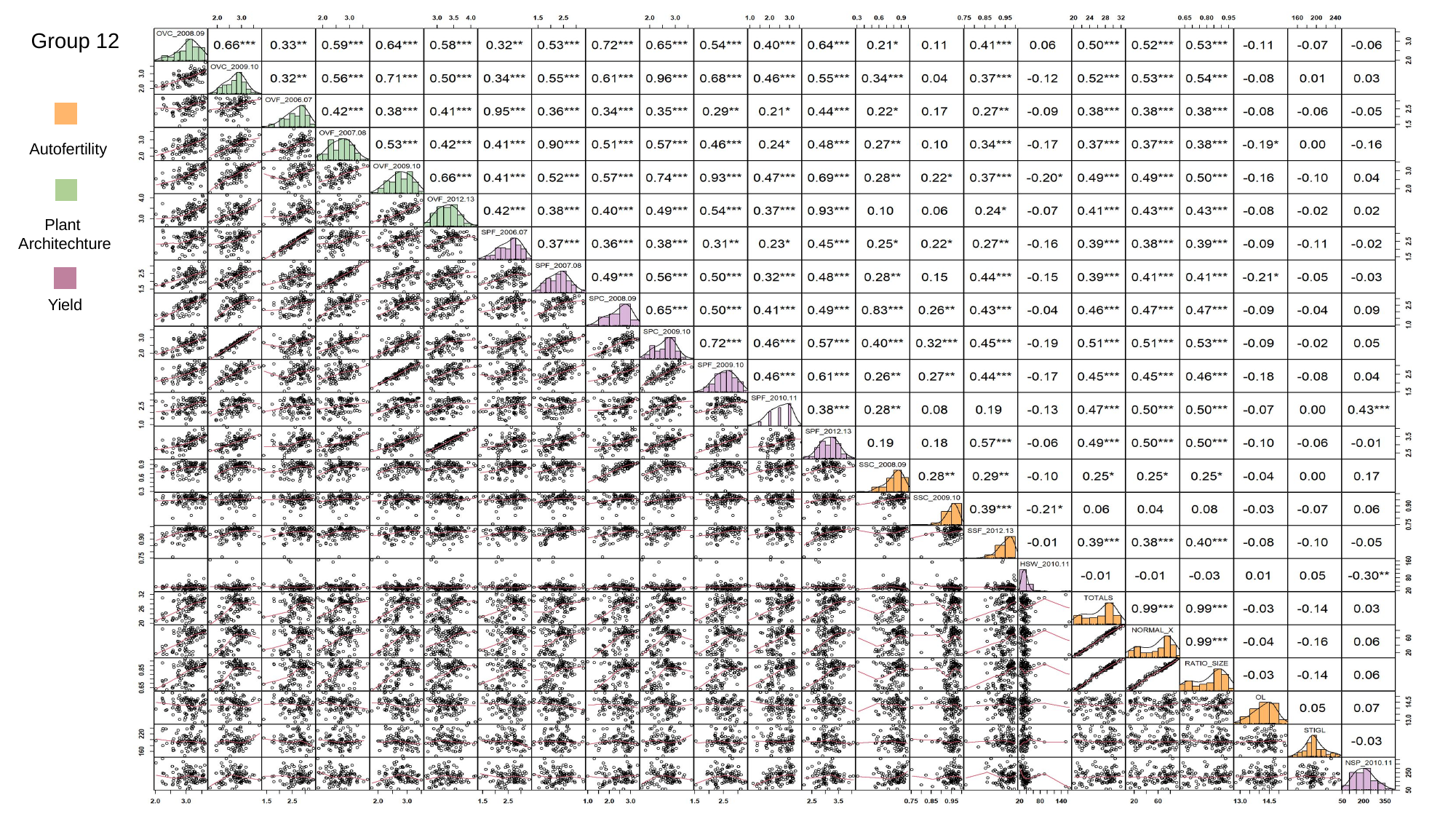

Group 12
Autofertility
Plant
Architechture
Yield
